# Comparison of rubella immunization rates in immigrant and Italian women of childbearing age: Results from the Italian behavioral surveillance system *PASSI* (2011-2015)

**DOI:** 10.1371/journal.pone.0178122

**Published:** 2017-10-02

**Authors:** Massimo Fabiani, Gianluigi Ferrante, Valentina Minardi, Cristina Giambi, Flavia Riccardo, Silvia Declich, Maria Masocco

**Affiliations:** 1 National Centre for Epidemiology, Surveillance and Health Promotion (CNESPS), Italian National Institute of Health (ISS), Rome, Italy; 2 European Programme for Intervention Epidemiology Training (EPIET), European Centre for Disease Prevention and Control (ECDC), Stockholm, Sweden; The University of Hong Kong, HONG KONG

## Abstract

**Background:**

International migration rapidly increased in the last decade, raising a renewed attention to its impact on public health. We evaluated differences in rubella immunization rate (RIR) between immigrant and Italian women of childbearing age and tried to identify the driving factors causing them.

**Methods:**

We analyzed data from the Italian behavioral surveillance system *PASSI* collected in 2011–2015 in a nationally representative sample of residents in Italy. The analysis was performed using log-binomial models to compare RIR between 41,094 Italian women and 3140 regular immigrant women of childbearing age (18–49 years), stratifying the latter by area of origin and length-of-stay in Italy (recent: ≤ 5-years; mid-term: 6-10-years; long-term: > 10-years).

**Results:**

Immigrant women showed a RIR of 36.0% compared to 60.2% among Italian women (RIR-ratio = 0.60, 95% confidence interval (CI): 0.57–0.63). Adjusting for demographic characteristics (i.e., sex, age and area of residence), socio-economic factors (i.e., education, occupation, family composition and economic status) and an indicator of the presence of at least one health-risk behavior (i.e., physical inactivity, current cigarette smoking, excessive alcohol consumption and excess weight) did not significantly change this difference (RIR-ratio = 0.56, 95% CI: 0.53–0.59). Recent immigrants (RIR-ratio = 0.47, 95% CI: 0.42–0.53) and immigrants from high migratory pressure countries (HMPC) in sub-Saharan Africa (RIR-ratio = 0.41, 95% CI: 0.31–0.56) and Asia (RIR-ratio = 0.42, 95% CI: 0.33–0.53) showed the greatest differences in RIR compared with Italian women.

**Conclusions:**

Differences in RIR between immigrant and Italian women were not explained by different demographic, socioeconomic and health-risk behaviors characteristics. As entitlement to free-of-charge immunization in Italy is universal, regardless of migration status, other informal barriers (e.g., cultural and barriers to information access) might explain lower RIRs in immigrant women, especially recent immigrants and those from HMPC in sub-Saharan Africa and Asia. Further investigations are needed to identify obstacles and appropriate promotion and access-enabling strategies for rubella immunization.

## Introduction

Worldwide migration is an increasing phenomenon; 244 million international migrants were estimated in 2015 (3.3% of the world’s population) [1]. Most migrants live in high-income countries (70.8%), accounting for 13% of the total population. Thirty-five million immigrants (6.9% of the population) were residing in the 28 European Union (EU) countries as of January 2015 [2]. In Italy, the number of regular immigrants formally residing in the country increased between 2005 and 2015 from 2.4 million (4.1% of the resident population) to 5.0 million (8.2% of the resident population) [3]. Of these, about 1.6 million (31.9%) were adult women of childbearing age (18–49 years).

Migrants are generally healthy when they arrive to Europe (“healthy migrant effect”) [4]. However, due to disadvantaged socio-economic conditions, cultural characteristics and reduced access to services for health prevention and care [5] compared with local populations, they can experience unsanitary living conditions in the host country that place them at increased risk for infectious diseases. For this reason, migrants are considered as one of the priority groups for the prevention and control of communicable diseases [6–8].

Vaccinations are among the most cost-effective strategies for the primary prevention of infectious diseases. As of today, vaccines have been licensed to prevent and control twenty-five different types of infection [9]. Among these, vaccines against diseases that can be transmitted vertically from pregnant women have an important role in terms of public health. When contracted by a woman in the early stages of pregnancy, rubella can infect the developing baby causing miscarriage, stillbirth or long-term sequelae (e.g., deafness, blindness, heart malformation and mental disability) [10]. It was estimated that fetal malformations due to congenital rubella occurred in 90% of newborns from women who were infected during the first 10 weeks of pregnancy [11].

Rubella immunization programs typically target both sexes at an early age in order to ensure high coverage among women when they reach reproductive maturity and reduce the risk of contact with infected persons during pregnancy. In Italy, rubella immunization is offered free-of-charge as part of the measles-mumps-rubella (MMR) combined vaccine in two doses: the first at 12–15 months of age and the second at 5–6 years of age [10,12]. Moreover, it is recommended to all susceptible adolescents and young adults. In particular, screening for rubella susceptibility and possible vaccination is recommended and offered free of charge to all women of childbearing age, with vaccination postponed in case of ongoing pregnancy.

Rubella immunization rates (RIR) among migrant populations hosted in European countries have been rarely documented. When this occurred, RIR was typically estimated among children and adolescents [13–16]. Information on rubella immunization rates in adult immigrants is still scarce, mostly based on relatively old data from restricted geographical settings [17–21]. This study aims to compare rubella immunization rates (RIR) in a nationally representative sample of immigrant and Italian women of childbearing age who were formally residing in Italy in 2011–2015, also trying to assess which were the driving factors causing differences.

## Materials and methods

### Data source and study population

*PASSI* (Progressi delle Aziende Sanitarie per la Salute in Italia—Progress by Local Health Units towards a Healthier Italy) is an ongoing nationwide surveillance system that, since 2008, monitors the prevalence of the major behavioral risk factors for non-communicable chronic diseases and the adherence to some important preventive measures among the Italian adult population (18–69 years of age). PASSI is jointly carried out by the Italian Local Health Units (LHUs) and regions, coordinated by the Italian National Institute of Health (ISS).

In each LHU, a proportionate random sample is drawn monthly from the enrolment list of residents stratified by sex and age group (18–34, 35–49, and 50–69 years). In particular, a random number generator is used to draw a simple random sample from each sex/age stratum proportionally to the stratum’s population size (proportionate allocation). Inclusion criteria are residence in the reference area of the LHU and availability of a telephone number; exclusion criteria are inability to be interviewed (e.g., serious handicaps) and being currently hospitalized or institutionalized. All the selected participants are informed in advance by letter about the purpose of the surveillance system and about confidentiality and protection of personal data (Italian legislative decree n. 196/2003). After provision of verbal consent, recorded in a dedicated section of the surveillance form, they are telephonically interviewed by specifically trained staff of the LHUs through a standardized questionnaire [22].

A total of 189,949 interviews were conducted in the period 2011–2015. The yearly response rate, calculated according to the American Association for Public Opinion Research RR4 standard [23], was always higher than 82%. Data on rubella immunization status were collected among 45,246 women of childbearing age (18–49 years). Of these, we analyzed data for 44,234 women with all information available for the analysis.

The *PASSI* surveillance system, including the consent procedure, was approved by the ethical committee of the Italian National Institute of Health (Prot. CE-ISS 06/158 dated 8 March 2007).

### Exposure, outcome and possible confounders

We assessed the association between citizenship (exposure) and rubella immunization status (outcome). Based on prior recommendations in literature [24], we analyzed data on foreign women who were regularly and formally resident in Italy (hereafter referred to as “immigrant women”) also stratifying them by macro-area of origin and length of stay in Italy. Macro-area of origin was classified according to citizenship and distinguishing between advanced development countries (ADC) with high gross national income (GNI) per capita and high migratory pressure countries (HMPC) with low/middle GNI per capita [25]. According to length of stay in Italy, immigrant women were categorized as recent (≤ 5 years), mid-term (6–10 years), or long-term (> 10 years). Rubella immunization status was classified and analyzed according to two categories: 1) immune women, i.e., women reporting to have been vaccinated or tested positive for rubella antibodies; 2) not immune women, i.e., susceptible women who were not vaccinated and were tested negative for rubella antibodies, or potentially susceptible women with unknown immunization status (no vaccination or unknown vaccination status, and test not done or unknown test result). We considered as possible confounders or mediating factors of the relationship between citizenship and rubella immunization the demographic characteristics (i.e., age and area of residence) and socio-economic conditions (i.e., education, occupation, household composition and self-perceived economic status), as well as the indicator variable of the presence of at least one of the following health-risk behaviors: physical inactivity (lack of intense and moderate physical activities during leisure time), current cigarette smoking, excessive alcohol consumption (binge drinking and/or regular assumption of 2 or more units per day), and excess weight (body mass index ≥ 25) (see S1 Appendix for details) [26].

### Statistical analysis

The demographic and socio-economic characteristics and the indicator of health-risk behaviors were described and compared between immigrant and Italian women using the chi-square test.

RIRs were calculated as the ratio of immune women to all women, including those with unknown immunization status (considered as potentially susceptible to rubella infection). We evaluated differences in RIR between immigrant and Italian women through log-binomial models, using rubella immunization rate ratios (RIR-ratio) and their 95% confidence intervals (CI) to describe the strength of the association between citizenship and RIR. To evaluate if and how different characteristics between immigrant and Italian women explain this association, we also ran stepwise multivariable models by firstly including the demographic variables, then adding socio-economic variables and lastly including the indicator variable of health-risk behaviors. We adopted the same stepwise approach to evaluate, separately for immigrant and Italian women, the association between RIR and any other factors. In this way, we avoided the over-adjustment for variables that are likely to play a mediating role in these relationships [27]. In detail, we assumed that demographic characteristics (first level) could partly explain socio-economic conditions (second level) that, in turn, could partly explain health-risk behaviors (third level). Based on this assumption, we presented RIR-ratios adjusted only for factors assigned to the same or preceding hierarchical levels. We also evaluated the effect modification according to citizenship by testing the interaction terms between citizenship and each factor included in the multivariable models through the adjusted Wald test.

In order to account for the sampling design, all the analyses were conducted using the Taylor series method for variance estimation and assigning each record a probability weight equal to the inverse of the sampling fraction in the corresponding LHU stratum. Absolute numbers are presented as they are counted in the sample while percentages and rate ratios are based on weighted data.

Statistical analysis was performed using Stata/MP version 13 (StataCorp LP, Texas, USA).

## Results

Immigrant women accounted for 6.0% of all women of childbearing age (Table 1). Most of them were citizens of HMPC in Europe (34.5% from EU countries and 29.3% from European countries outside EU) (Table 1). Recent immigrants accounted for about one-fifth of all immigrant women (22.2%), while mid-term and long-term immigrants accounted for 40.0% and 37.7%, respectively.

**Table 1:** Demographic, socio-economic and behavioral characteristics of Italian and immigrant women of childbearing age (Italy, 2011–2015).

	Italian women and all immigrant women			Immigrant women by length of stay in Italy				Immigrant women by area of origin							
								ADC	HMPC						
	Italian women	Immigrant women	p-value	≤ 5 years	6–10 years	> 10 years	p-value	All	European Union (EU)	Europe outside EU	North Africa	sub-Saharan Africa	Asia	America	p-value
	n (%)^a^	n (%)^a^		n (%)^a^	n (%)^a^	n (%)^a^		n (%)^a^	n (%)^a^	n (%)^a^	n (%)^a^	n (%)^a^	n (%)^a^	n (%)^a^	
**Age group**			< 0.001				< 0.001								< 0.001
18–24 years	6819 (17.3)	402 (11.3)		169 (20.1)	137 (9.9)	96 (7.6)		3 (2.0)	105 (9.2)	160 (14.5)	55 (16.3)	11 (6.9)	27 (10.4)	41 (12.2)	
25–34 years	10,899 (26.8)	1149 (36.5)		350 (45.2)	532 (43.5)	267 (23.9)		17 (13.0)	417 (38.7)	357 (37.0)	113 (38.4)	60 (39.6)	96 (40.6)	89 (30.3)	
35–49 years	23,376 (55.9)	1589 (52.2)		256 (34.8)	564 (46.6)	769 (68.5)		105 (85.0)	570 (52.1)	438 (48.5)	119 (45.3)	78 (53.5)	101 (48.9)	178 (57.5)	
**Area of residence**			< 0.001				0.652								< 0.001
North	17,730 (33.7)	1793 (53.6)		444 (52.9)	716 (54.6)	633 (53.0)		68 (48.9)	509 (42.0)	591 (58.8)	201 (68.0)	114 (77.3)	123 (48.0)	187 (60.6)	
Centre	10,918 (22.8)	1129 (35.1)		264 (33.9)	440 (34.6)	425 (36.4)		47 (38.6)	495 (45.5)	290 (28.5)	72 (24.2)	29 (17.7)	84 (37.3)	112 (33.5)	
South and islands	12,446 (43.5)	218 (11.2)		67 (13.2)	77 (10.8)	74 (10.6)		10 (12.5)	88 (12.4)	74 (12.7)	14 (7.8)	6 (5.0)	17 (14.7)	9 (5.9)	
**Educational level**			< 0.001				< 0.001								< 0.001
Low (≤ 8 years)	10,412 (25.5)	1240 (37.7)		353 (44.7)	483 (37.7)	404 (33.7)		17 (13.9)	295 (25.8)	416 (41.6)	178 (63.5)	86 (53.6)	132 (56.1)	116 (32.7)	
Medium (9–13 years)	21,972 (53.0)	1486 (48.6)		309 (40.9)	591 (48.0)	586 (53.9)		55 (45.4)	690 (64.5)	392 (42.5)	84 (27.3)	44 (34.1)	65 (30.7)	156 (52.9)	
High (> 13 years)	8710 (21.4)	414 (13.6)		113 (14.4)	159 (14.3)	142 (12.4)		53 (40.6)	107 (9.7)	147 (15.8)	25 (9.2)	19 (12.2)	27 (13.2)	36 (14.3)	
**Occupational status**			0.229				< 0.001								< 0.001
Employed	26,275 (60.3)	1833 (58.9)		378 (48.2)	726 (58.5)	729 (65.7)		86 (69.4)	763 (68.8)	522 (55.6)	81 (26.9)	71 (50.4)	109 (51.9)	201 (66.7)	
Unemployed	14,819 (39.7)	1307 (41.1)		397 (51.8)	507 (41.5)	403 (34.3)		39 (30.6)	329 (31.2)	433 (44.4)	206 (73.1)	78 (49.6)	115 (48.1)	107 (33.3)	
**Household composition**			< 0.001				< 0.001								< 0.001
Alone	2667 (6.0)	213 (6.5)		70 (9.0)	83 (5.8)	60 (5.7)		8 (5.9)	91 (8.4)	57 (5.4)	11 (3.2)	14 (7.7)	9 (4.6)	23 (6.9)	
Only partner	6045 (14.1)	550 (17.2)		175 (21.9)	207 (17.4)	168 (14.4)		27 (21.4)	249 (20.7)	135 (14.7)	31 (10.8)	21 (14.5)	27 (16.0)	60 (18.8)	
Partner with kids^b^	12,747 (30.2)	1265 (40.2)		267 (34.3)	506 (41.4)	492 (42.5)		62 (49.7)	370 (33.8)	397 (42.4)	163 (56.9)	62 (40.7)	114 (49.4)	97 (30.6)	
Partner with others	4252 (10.6)	314 (10.3)		49 (5.7)	117 (10.1)	148 (13.1)		15 (12.6)	106 (10.5)	113 (11.7)	18 (6.8)	13 (9.1)	26 (11.1)	23 (7.2)	
Others without partner	15,383 (39.1)	798 (25.8)		214 (29.0)	320 (25.3)	264 (24.4)		13 (10.3)	276 (26.6)	253 (25.8)	64 (22.3)	39 (27.9)	48 (18.8)	105 (36.5)	
**Economic conditions**			< 0.001				0.826								< 0.001
Non adequate	22,769 (59.0)	2292 (73.7)		563 (74.3)	907 (74.0)	822 (73.0)		56 (45.1)	743 (70.4)	721 (74.3)	240 (85.9)	125 (85.5)	162 (75.7)	245 (77.1)	
Adequate	18,325 (41.0)	848 (26.3)		212 (25.7)	326 (26.0)	310 (27.0)		69 (54.9)	349 (29.6)	234 (25.7)	47 (14.1)	24 (14.5)	62 (24.3)	63 (22.9)	
**Risky behaviors**^c^			0.477				0.832								0.009
No	16,710 (38.8)	1247 (39.6)		322 (40.5)	492 (39.7)	433 (38.9)		56 (45.1)	400 (36.2)	418 (42.6)	89 (29.2)	58 (44.1)	95 (44.3)	131 (42.5)	
Yes	24,384 (61.2)	1893 (60.4)		453 (59.5)	741 (60.3)	699 (61.1)		69 (54.9)	692 (63.8)	537 (57.4)	198 (70.8)	91 (55.9)	129 (55.7)	177 (57.5)	
**Total**	41,094 (94.0)	3140 (6.0)		775 (22.2)	1233 (40.0)	1132 (37.7)		125 (4.2)	1092 (34.5)	955 (29.3)	287 (8.2)	149 (5.2)	224 (8.3)	308 (10.4)	

ADC, advanced development countries; HMPC, high migratory pressure countries.

^a^ Absolute numbers are presented as they are counted in the sample while percentages are based on weighted data (each record was assigned a probability weight equal to the inverse of the sampling fraction in the corresponding LHU stratum).

^b^ Children ≤ 14 years.

^c^ Presence of at least one of the following health-risk behaviors: inactivity (lack of intense and moderate physical activities during leisure time), current cigarette smoking, excessive alcohol consumption (binge drinking and/or regular assumption of 2 or more units per day), overweight (body mass index ≥ 25).

### Demographic and socio-economic characteristics and health-risk behaviors

The demographic characteristics and socio-economic conditions of immigrant women were significantly different from those of Italian women (Table 1). Immigrant women were more frequently between 25 and 34 years of age (36.5% *vs* 27.8%; P < 0.001) and resided more frequently in north-central Italy (88.7% *vs* 56.5%; P < 0.001). They had a lower level of education (37.7% *vs* 25.5% did not receive secondary education; P < 0.001) and reported more frequently than Italian women to live with a partner and children less than 14 years of age (40.2% *vs* 30.2%; P < 0.001). They also reported more frequently economic difficulties (73.7% *vs* 59.0%; P < 0.001), while no significant differences in employment rate and health-risk behaviors were observed between the two groups.

Recent immigrants were younger (65.3% *vs* 31.5% were less than 35 years of age; P < 0.001), less educated (44.7% *vs* 33.7% did not receive secondary education; P < 0.001), and less frequently employed (48.2% *vs* 65.7%; P < 0.001) than long-term immigrants. They also reported less frequently than long-term immigrants to live in large households with a partner and other family members (40.0% *vs* 55.6%; P < 0.001). We also observed significant differences in demographic and socio-economic characteristics among immigrant women from different geographical areas. More specifically, compared with other immigrant women, particularly those from ADC, women from African HMPC reported worse socio-economic conditions.

### Rubella immunization rates

More than one-third of women were not aware of their rubella immunization status (36.6%; 56.8% in immigrant women compared to 35.3% in Italian women) (Table 2). Among women with known immunization status, rubella susceptibility was 7.3% (16.6% and 6.9% in immigrant and Italian women, respectively).

**Table 2:** Rubella immunization status of Italian and immigrant women of childbearing age (Italy, 2011–2015).

	Immune		Not immune	
	Vaccinated	Tested positive^a^	Susceptible^b^	Unknown^c^
	n (%)^d^	n (%)^d^	n (%)^d^	n (%)^d^
**Italian women**	17,795 (40.4)	7795 (19.9)	1753 (4.4)	13,751 (35.3)
**Immigrant women**	797 (25.2)	346 (10.8)	207 (7.2)	1790 (56.8)
Length of stay in Italy: ≤ 5 years	171 (22.1)	63 (7.7)	47 (6.4)	494 (63.8)
Length of stay in Italy: 6–10 years	282 (22.0)	148 (11.2)	90 (8.0)	713 (58.9)
Length of stay in Italy: > 10 years	344 (30.5)	135 (12.3)	70 (6.8)	583 (50.4)
ADC	59 (47.0)	21 (18.0)	2 (1.3)	43 (33.7)
HMPC—European Union (EU)	267 (24.5)	120 (10.7)	73 (7.5)	632 (57.3)
HMPC—Europe outside EU	264 (27.1)	100 (10.4)	56 (5.7)	535 (56.8)
HMPC—northern Africa	64 (22.8)	38 (12.7)	28 (8.8)	157 (55.7)
HMPC—sub-Saharan Africa	28 (21.7)	10 (5.6)	17 (11.8)	94 (60.9)
HMPC—Asia	31 (13.6)	27 (13.2)	17 (6.8)	149 (66.3)
HMPC—America	84 (26.4)	30 (8.7)	14 (9.2)	180 (55.7)
**Total**	18,592 (39.5)	8141 (19.3)	1960 (4.6)	15,541 (36.6)

ADC, advanced development countries; HMPC, high migratory pressure countries.

^a^ Women reporting to have not been vaccinated but tested positive for rubella antibodies.

^b^ Women reporting to have not been vaccinated and tested negative for rubella antibodies.

^c^ Women reporting to have not been vaccinated or unknown vaccination status, and test not done or unknown test result (potentially susceptible).

^d^ Absolute numbers are presented as they are counted in the sample while percentages are based on weighted data (each record was assigned a probability weight equal to the inverse of the sampling fraction in the corresponding LHU stratum).

RIR in all women of childbearing age was 58.8%, significantly lower in immigrant women compared to Italian women (36.0% *vs* 60.2%; RIR-ratio = 0.60, 95% CI: 0.57–0.63) (Table 3). After adjusting for demographic and socio-economic characteristics and for the indicator variable of health-risk behaviors, this difference did not significantly change (RIR-ratio = 0.56, 95% CI: 0.53–0.59).

**Table 3:** Crude and adjusted ratios of rubella immunization rates in immigrant compared with Italian women of childbearing age (Italy, 2011–2015).

	Not immune	Immune^a^	RIR-ratio^b^	RIR-ratio^c^	RIR-ratio^d^	RIR-ratio^e^
	n (%)^f^	n (%)^f^	(95% CI)^f^	(95% CI)^f^	(95% CI)^f^	(95% CI)^f^
**Italian women**^g^	15,504 (39.8)	25,590 (60.2)	1	1	1	1
**All immigrant women**	1997 (64.0)	1143 (36.0)	0.60 (0.57–0.63)	0.56 (0.53–0.59)	0.56 (0.53–0.59)	0.56 (0.53–0.59)
Length of stay: ≤ 5 years	541 (70.2)	234 (29.8)	0.49 (0.44–0.56)	0.47 (0.41–0.53)	0.47 (0.42–0.53)	0.47 (0.42–0.53)
Length of stay: 6–10 years	803 (66.9)	430 (33.1)	0.55 (0.50–0.60)	0.51 (0.47–0.56)	0.51 (0.47–0.56)	0.51 (0.47–0.56)
Length of stay: > 10 years	653 (57.2)	479 (42.8)	0.71 (0.66–0.77)	0.66 (0.61–0.71)	0.65 (0.60–0.70)	0.65 (0.60–0.70)
**ADC**	45 (35.0)	80 (65.0)	1.08 (0.94–1.24)	1.00 (0.87–1.15)	0.96 (0.85–1.09)	0.96 (0.85–1.09)
Length of stay: ≤ 5 years	8 (46.7)	11 (53.3)	0.89 (0.55–1.42)	0.83 (0.52–1.33)	0.80 (0.51–1.27)	0.82 (0.52–1.30)
Length of stay: 6–10 years	9 (33.3)	16 (66.7)	1.11 (0.82–1.49)	1.04 (0.77–1.39)	1.03 (0.79–1.33)	1.02 (0.79–1.33)
Length of stay: > 10 years	28 (33.)	53 (66.8)	1.11 (0.94–1.31)	1.02 (0.87–1.21)	0.98 (0.85–1.12)	0.97 (0.84–1.12)
**HMPC—European Union (EU)**	705 (64.8)	387 (35.2)	0.58 (0.53–0.64)	0.55 (0.50–0.61)	0.56 (0.51–0.62)	0.56 (0.51–0.62)
Length of stay: ≤ 5 years	191 (72.5)	75 (27.5)	0.46 (0.36–0.58)	0.44 (0.35–0.55)	0.47 (0.37–0.59)	0.47 (0.37–0.59)
Length of stay: 6–10 years	321 (68.0)	163 (32.0)	0.53 (0.46–0.62)	0.50 (0.43–0.58)	0.51 (0.44–0.59)	0.51 (0.44–0.59)
Length of stay: > 10 years	193 (55.3)	149 (44.7)	0.74 (0.64–0.85)	0.69 (0.60–0.79)	0.68 (0.60–0.78)	0.68 (0.60–0.78)
**HMPC—Europe outside EU**	591 (62.5)	364 (37.5)	0.62 (0.56–0.69)	0.58 (0.53–0.64)	0.57 (0.52–0.63)	0.57 (0.52–0.63)
Length of stay: ≤ 5 years	158 (64.9)	82 (35.1)	0.58 (0.47–0.62)	0.55 (0.45–0.68)	0.55 (0.45–0.67)	0.55 (0.45–0.67)
Length of stay: 6–10 years	241 (65.6)	135 (34.4)	0.57 (0.49–0.67)	0.53 (0.45–0.62)	0.52 (0.45–0.61)	0.52 (0.44–0.61)
Length of stay: > 10 years	192 (57.8)	147 (42.2)	0.70 (0.61–0.81)	0.65 (0.57–0.76)	0.64 (0.56–0.74)	0.64 (0.56–0.74)
**HMPC—northern Africa**	185 (64.5)	102 (35.5)	0.59 (0.49–0.70)	0.54 (0.45–0.64)	0.51 (0.43–0.61)	0.52 (0.43–0.62)
Length of stay: ≤ 5 years	57 (76.3)	20 (23.7)	0.39 (0.25–0.62)	0.37 (0.23–0.58)	0.35 (0.23–0.55)	0.35 (0.23–0.55)
Length of stay: 6–10 years	68 (57.7)	46 (42.3)	0.70 (0.55–0.90)	0.64 (0.50–0.82)	0.61 (0.48–0.78)	0.62 (0.49–0.78)
Length of stay: > 10 years	60 (62.6)	36 (37.4)	0.62 (0.46–0.84)	0.56 (0.42–0.75)	0.53 (0.40–0.72)	0.53 (0.40–0.72)
**HMPC—sub-Saharan Africa**	111 (72.7)	38 (27.3)	0.45 (0.33–0.62)	0.41 (0.33–0.54)	0.41 (0.31–0.56)	0.41 (0.31–0.56)
Length of stay: ≤ 5 years	24 (72.3)	10 (27.7)	0.46 (0.26–0.81)	0.43 (0.24–0.75)	0.44 (0.25–0.77)	0.44 (0.25–0.76)
Length of stay: 6–10 years	42 (83.0)	9 (17.0)	0.28 (0.14–0.58)	0.26 (0.13–0.52)	0.25 (0.13–0.52)	0.25 (0.12–0.51)
Length of stay: > 10 years	45 (63.6)	19 (36.4)	0.60 (0.41–0.90)	0.54 (0.36–0.80)	0.55 (0.37–0.81)	0.55 (0.38–0.81)
**HMPC—Asia**	166 (73.1)	58 (26.9)	0.45 (0.34–0.58)	0.42 (0.33–0.54)	0.42 (0.33–0.53)	0.42 (0.33–0.53)
Length of stay: ≤ 5 years	53 (73.3)	18 (26.7)	0.44 (0.29–0.67)	0.42 (0.28–0.63)	0.39 (0.26–0.60)	0.39 (0.26–0.60)
Length of stay: 6–10 years	55 (76.4)	20 (23.6)	0.39 (0.24–0.65)	0.38 (0.24–0.61)	0.39 (0.25–0.61)	0.39 (0.25–0.61)
Length of stay: > 10 years	58 (69.5)	20 (30.5)	0.51 (0.34–0.74)	0.46 (0.31–0.67)	0.46 (0.32–0.67)	0.46 (0.32–0.67)
**HMPC—America**	194 (64.9)	114 (35.1)	0.58 (0.49–0.70)	0.54 (0.45–0.64)	0.55 (0.46–0.66)	0.55 (0.46–0.66)
Length of stay: ≤ 5 years	50 (73.8)	18 (26.2)	0.44 (0.28–0.68)	0.40 (0.26–0.62)	0.40 (0.26–0.62)	0.40 (0.26–0.62)
Length of stay: 6–10 years	67 (65.3)	41 (34.7)	0.58 (0.42–0.78)	0.53 (0.39–0.72)	0.55 (0.40–0.74)	0.55 (0.40–0.74)
Length of stay: > 10 years	77 (60.9)	55 (39.1)	0.65 (0.50–0.84)	0.60 (0.46–0.77)	0.62 (0.49–0.79)	0.62 (0.49–0.79)

RIR, rubella immunization rate; CI, confidence interval; ADC, advanced development countries; HMPC, high migratory pressure countries.

^a^ Women reporting to have been vaccinated or tested positive for rubella antibodies.

^b^ Crude RIR-ratio.

^c^ RIR-ratio adjusted for age and area of residence.

^d^ RIR-ratio adjusted for age, area of residence, educational level, occupational status, household composition, and economic resources.

^e^ RIR-ratio adjusted for age, area of residence, educational level, occupational status, household composition, economic resources, and health-risk behaviors.

^f^ Absolute numbers are presented as they are counted in the sample while percentages and rate ratios are based on weighted data (each record was assigned a probability weight equal to the inverse of the sampling fraction in the corresponding LHU stratum).

^g^ Reference category for all RIR-ratios presented in the table.

Compared with Italian women, after adjustment, the greatest differences in RIR were observed among recent immigrants (RIR-ratio = 0.47, 95% CI: 0.42–0.53) and among immigrants from HMPC in sub-Saharan Africa (RIR-ratio = 0.41, 95% CI: 0.31–0.56) and Asia (RIR-ratio = 0.42, 95% CI: 0.33–0.53). No difference was observed between Italian women and immigrant women from ADC (RIR-ratio = 0.96, 95% CI: 0.85–1.09). An increase in RIR from 29.8% in recent immigrants (≤ 5 years in Italy) to 42.8% in long-term immigrants (> 10 years in Italy) was observed. After adjustment, the difference in RIR with Italian women was found to decrease with length of stay in Italy, both overall and by area of origin, although this was less evident among immigrant women from HMPC in Europe (outside EU), sub-Saharan Africa and Asia.

Immigrant and Italian women showed some differences in the levels of association between rubella immunization and the other factors considered in this analysis (Table 4). In particular, the two groups showed a different profile according to age (interaction test, P = 0.002), educational level (interaction test, P = 0.002) and household composition (interaction test, P = 0.043). Increased age was associated with immunization among Italian women, while it was not among immigrants. Increased level of education and living in large households, especially those including a partner and children less than 14 years of age, were associated with immunization in both groups, but the strength of these associations was significantly higher among immigrant women. In both groups, rubella immunization was associated with living in northern Italy, while no relevant associations with occupational status, economic resources and health-risk behaviors were observed.

**Table 4:** Factors associated with rubella immunization in Italian and immigrant women of childbearing age (Italy, 2011–2015).

	Italian women			Immigrant women		
	Not immune	Immune	RIR-ratio^a^	Not immune	Immune	RIR-ratio^a^
	n (%)^b^	n (%)^b^	(95% CI)^b^	n (%)^b^	n (%)^b^	(95% CI)^b^
**Age group (1)**^c^						
18–24 years	2715 (41.7)	4104 (58.3)	1	256 (63.0)	146 (37.0)	1
25–34 years	4301 (41.7)	6598 (58.3)	1.00 (0.97–1.04)	688 (60.2)	461 (39.8)	1.08 (0.91–1.28)
35–49 years	8488 (38.2)	14,888 (61.8)	1.05 (1.02–1.08)	1053 (66.8)	536 (33.2)	0.92 (0.78–1.08)
**Area of residence (1)**						
North	5495 (31.3)	12,235 (68.7)	1	1105 (61.1)	688 (38.9)	1
Centre	4005 (37.3)	6913 (62.7)	0.91 (0.89–0.93)	737 (65.1)	392 (34.9)	0.91 (0.81–1.02)
South and islands	6004 (47.6)	6442 (52.4)	0.76 (0.75–0.78)	155 (74.0)	63 (26.0)	0.68 (0.52–0.88)
**Educational level (2)**^c^						
Low (≤ 8 years)	4334 (44.0)	6078 (56.0)	1	851 (69.0)	389 (31.0)	1
Medium (9–13 years)	8101 (39.1)	13,871 (60.9)	1.09 (1.06–1.11)	915 (62.5)	571 (37.5)	1.30 (1.15–1.47)
High (> 13 years)	3069 (36.4)	5641 (63.6)	1.14 (1.11–1.17)	231 (55.5)	183 (44.5)	1.52 (1.30–1.77)
**Occupational status (2)**						
Employed	9446 (37.8)	16,829 (62.2)	1	1214 (66.1)	619 (33.9)	1
Unemployed	6058 (42.8)	8761 (57.2)	0.99 (0.97–1.01)	783 (61.0)	524 (39.0)	1.05 (0.94–1.17)
**Household composition (2)**^c^						
Alone	1485 (56.0)	1182 (44.0)	1	171 (79.5)	42 (20.5)	1
Only partner	2707 (45.8)	3338 (54.2)	1.27 (1.19–1.35)	370 (69.8)	180 (30.2)	1.43 (1.02–2.00)
Partner with kids^d^	2895 (24.5)	9852 (75.5)	1.78 (1.69–1.88)	678 (53.1)	587 (46.9)	2.26 (1.66–3.08)
Partner with others	1695 (42.2)	2557 (57.8)	1.45 (1.36–1.55)	227 (72.1)	87 (27.9)	1.41 (0.97–2.03)
Others without partner	6722 (46.2)	8661 (53.8)	1.25 (1.18–1.32)	551 (70.0)	247 (30.0)	1.44 (1.04–1.99)
**Economic conditions (2)**						
Non adequate	9067 (41.8)	13,702 (58.2)	1	1471 (64.6)	821 (35.4)	1
Adequate	6437 (36.9)	11,888 (63.1)	1.00 (0.98–1.02)	526 (62.)	322 (37.9)	1.01 (0.89–1.13)
**Risky behaviours (3)**						
No	5772 (36.4)	10,938 (63.6)	1	778 (62.9)	469 (37.1)	1
Yes	9732 (41.9)	14,652 (58.1)	0.96 (0.94–0.98)	1219 (64.7)	674 (35.3)	0.97 (0.87–1.08)

RIR-ratio, immunization rate ratio; CI, confidence interval; ADC, advanced development countries; HMPC, high migratory pressure countries.

Numbers in parentheses near the variable names indicate the hierarchical level assigned to each factor in multivariable analysis (from 1 to 3).

^a^ RIR-ratio adjusted for all the factors assigned to the same hierarchical level or the previous ones.

^b^ Absolute numbers are presented as they are counted in the sample while percentages and rate ratios are based on weighted data (each record was assigned a probability weight equal to the inverse of the sampling fraction in the corresponding LHU stratum).

^c^ Statistically significant interaction with citizenship according to the adjusted Wald test (P < 0.05).

^d^ Children ≤ 14 years.

## Discussion

Immigrant women showed a significantly different demographic and socio-economic profile compared to Italian women, while health-risk behaviors did not significantly differ between the two groups. We also observed differences in demographic characteristics and socio-economic conditions within immigrants according to length of stay in Italy and area of origin. Recent immigrants and those from HMPC in Africa appeared more disadvantaged compared to long-term immigrants and those from other geographical areas. This finding is consistent with estimates from other European countries, where African migrants were found to be less educated and more disadvantaged in the labor market than migrants from other continents [28–31]. By contrast, as expected, the socio-economic conditions of immigrants from ADC appeared much better than those of immigrants from HMPC, and also better than those observed among Italian women.

Overall, excluding women with unknown immunization status, the percentage of susceptible women was 7.3%; 16.6% among immigrants compared to 6.9% among Italian women. These rates are still above the maximum susceptibility rate of 5% defined by the Italian Ministry of Health in the national plan for the elimination of measles and congenital rubella [12]. This finding is consistent with those from other local studies conducted in Italy, where the percentage of women of childbearing age at risk of rubella infection among those tested for rubella antibodies was found to vary from 11.7% to 17.8% among immigrant women [17,18] and estimated at 6.2% among Italian women [18]. This is also consistent with findings from the most recent studies conducted in other European countries, where rubella susceptibility was found to be 5.9% in Catalonia (Spain) and 6.3% in Liverpool (UK) [19,32], with higher rates observed in immigrant women [19–21].

Consistently with estimates from the Sicily region in southern Italy, where 44.8% of pregnant women reported no screening before their current pregnancy [33], we found that about 40% of the women included in our study were unaware of their rubella immunization status, almost all of them because they were never tested (> 95% in both immigrant and Italian women). This finding suggests that the utilization of the rubella screening service is still low, particularly among immigrant women, who showed a higher rate of unawareness compared to Italian women. There are no formal access barriers to the Italian screening service. It is offered free of charge by the Italian national health system with no entitlement restrictions linked with citizenship. This suggests that its underuse might be due to a low risk perception about congenital rubella that needs be addressed through effective risk communication campaigns.

The estimation of RIRs and the analysis of factors associated with differences in RIR between immigrant and Italian women were carried out including women with unknown immunization status (no vaccination or unknown vaccination status, and test not done or unknown test result). This was because we considered these women as potentially susceptible to rubella infection and therefore a target group for prevention interventions (screening and possible vaccination). Consistently with susceptibility (calculated excluding women with unknown immunization status), immigrant women showed a reduced RIR compared to Italian women (36.0% *vs* 60.2%), especially recent immigrants and those from HMPC in sub-Saharan Africa and Asia. Recent immigrants are likely to be less informed about screening opportunities and, when coming from countries where rubella screening is not routinely implemented, they are also likely to have been less exposed to rubella immunization compared with long-term immigrants. Differences by geographical area of origin are in line with findings from Spain [19], United Kingdom [21], and the Sicily region of Italy [17], where decreased RIRs were observed in immigrant women from Africa and Asia. In general, our results are also consistent with findings from other European studies that compared RIR between immigrant children and adolescents and same-age national peers, all showing reduced immunization rates in immigrants [13–16].

In our study, the different demographic, socio-economic and health-risk behaviors profile did not explain the reduced RIR in immigrant women compared to Italian women. In particular, we found that, after adjustment for these factors, the difference in RIR with Italian women remained more pronounced for recent immigrants and immigrants from HMPC in sub-Saharan Africa and Asia, independently on their length of stay in Italy. Regardless of citizenship, entitlement to free screening and immunization is equivalent for all residents in Italy, thus suggesting that other informal barriers to accessing screening and immunization services disproportionally affect immigrant women, especially those in these sub-groups. Informal barriers that could play a relevant role include cultural barriers and barriers to information access. Immigrants often face challenges when trying to access routine vaccination services. In part, this could be due to an information gap: immigrants could be unaware of these services or be unaware of entitlement and gratuity. Moreover, it could also be due to unwillingness to use services for cultural, religious or other reasons [6,34]. This is why providing culturally sensitive information, training health professionals in culturally competent service delivery, and engaging key individuals from the migrant community to promote immunization could be important to meet health needs of immigrants and overcome informal barriers to immunization [34–38].

The analysis of factors associated with rubella immunization in immigrant and Italian women showed some differences between these groups. The slight association between age and rubella immunization that we observed among Italian women is likely to reflect the increasing exposure to rubella antenatal screening and post-partum vaccination with age. In fact, compared to younger women, older women are more likely to have experienced a pregnancy and to have therefore accessed these preventive services. This could not have been the case for immigrant women from countries where rubella antenatal screening and post-partum vaccination are not routinely implemented. An increased level of education, as well as living in large households, was found to be associated with rubella immunization, especially among immigrant women. This result is consistent with a previous study conducted in Spain that showed a positive association between parental education and primary vaccination of children born from immigrants [14]. Women living with a partner and children are very likely to have experienced a pregnancy that, in turn, could have favored their access to rubella screening and vaccination. Moreover, contacts with services for children’s immunization and sensitivity campaigns for the prevention of congenital rubella could have induced these women to look for protection before a new pregnancy.

Our study presents some limitations. Firstly, it only included foreign people formally residing in Italy. It did not include irregular migrants and regular migrants with no formal residence, who, according to recent estimates, accounted respectively for about 6% and 7% of all migrants in Italy [39,40]. RIR in these sub-groups is probably reduced compared with RIR in regular immigrants formally residing in the country. Even though there are no legal impediments to entitlement, gratuity and anonymous access to health services in relation to migrant status, it is likely that they are less aware of entitlement rights and more fearful of being identified by national authorities [5]. Another limitation of our study is that we estimated RIRs based on self-reported immunization status. These estimates could have been affected by recall and social desirability biases [41,42], leading to possible overestimation of immunization rates, in particular among immigrants [41]. In this case, the difference in RIR between immigrant and Italian women might have been underestimated.

However, this study has also some strengths. Firstly, the large sample size guaranteed an adequate statistical power to detect relevant differences as statistically significant. Secondly, the demographic characteristics of our sample (i.e., distribution of age and area of residence by citizenship) well reflected those of the country’s reference population for the same time-period [3], thus suggesting a good level of representativeness. Finally, although we aimed to estimate rubella immunization rather than rubella incidence, we performed the analysis taking into account a multidimensional framework that was found to comprehensively describe risk factors for infectious diseases in migrant populations [43]. In our knowledge, only two studies have been previously conducted in Italy to investigate rubella immunization in immigrant women [17,18]. Both of them were carried out in restricted geographical settings on relatively small samples. We have tried to fill in this information gap presenting recent estimates that are based on a large sample from the whole country’s resident population.

## Conclusions

Immigrant women showed a reduced RIR compared to Italian women, especially recent immigrants and those from HMPC in sub-Saharan Africa and Asia. This difference was not explained by the different demographic, socioeconomic and health-risk behaviors profile between the two groups. As entitlement to screening and immunization services in Italy is universal and free-of-charge for all people in the country, regardless of their citizenship and migration status, other informal barriers (e.g., cultural and barriers to information access) might explain lower RIRs in immigrant women. These findings could guide further studies, both qualitative and quantitative, aimed at identifying obstacles and appropriate promotion and access-enabling strategies for rubella immunization in this vulnerable population.

## Supporting information

S1 Appendix Assessment modalities of self-perceived economic status and health-risk behaviors. (DOCX)
