# Supplementary material for: Comparison of rubella immunization rates in immigrant and Italian women of childbearing age: Results from the Italian behavioral surveillance system PASSI (2011-2015)
Source: PLoS One. 2017 Oct 2;12(10):e0178122. doi: 10.1371/journal.pone.0178122 (PMC5624576; doi:10.1371/journal.pone.0178122)
Supplement: S1 Appendix — (DOCX) [file pone.0178122.s001.docx]

**S1 Appendix**

**Assessment modalities of self-perceived economic status and health-risk behaviors**

Self-perceived economic status

Self-perceived economic status was assessed asking respondents how they get to the end of the month with the available economic resources. Those who reported some or no difficulties at all were classified in the group of “adequate economic condition”; those who reported many difficulties were classified in the group of “non adequate economic condition”.

Physical inactivity in the leisure time

Leisure-time physical activity is explored by asking respondents if in the 30 days before the interview they have engaged in moderate physical activity (vacuuming, gardening, brisk walking, or bicycling) or vigorous physical activity (running, aerobics, or heavy yard work), without considering working time; additionally, for both levels of physical activity, they were asked for how many days in a week and for how many minutes in those days they exercised, on average. The products of the latter 2 values were obtained for each level of physical activity; then an overall measure of weekly physical activity was obtained by doubling the minutes of weekly vigorous physical activity and adding this value to the minutes of weekly moderate physical activity, because 1 minute of vigorous physical activity is assumed to be equivalent to 2 minutes of moderate physical activity [1]. Daily physical activity bouts of less than 10 minutes’ duration were not included in the calculation. Respondents were considered inactive (no leisure time physical activity), if the weekly minutes of physical activity were less than 10.

Current cigarette smoking

Current smokers were defined as those who report having smoked at least 100 cigarettes in their lifetime and being current smokers on every day or some days when interviewed.

Excessive alcohol consumption

Men were considered to be excessive alcohol consumers if they drank more than 2 alcoholic units (AUs) per day on average or if they reported having 5 or more AUs during a single occasion in the 30 days before the interview. For women, the threshold was more than 1 AU per day on average or 4 or more AUs during a single occasion. One AU corresponds to a glass of wine (125 mL), a can of beer (330 mL), or 1 shot glass of spirits (40 mL) [2].

Excess weight

Nutritional status was assessed through the Body Mass Index (BMI), calculated from self-reported weight and height (kg/m2). Respondents were defined as overweight if their BMI was equal or greater than 25.

1. World Health Organization. Global recommendations on physical activity for health. 2010. http://whqlibdoc.who.int/ publications/2010/9789241599979_eng.pdf.
2. Center for Disease Control and Prevention. Alcohol and public health; 2015.

http://www.cdc.gov/alcohol/ faqs.htm#excessive.
